# Cerium dioxide nanoparticles exacerbate house dust mite induced type II airway inflammation

**DOI:** 10.1186/s12989-018-0261-5

**Published:** 2018-05-23

**Authors:** Kirsty Meldrum, Sarah B. Robertson, Isabella Römer, Tim Marczylo, Lareb S. N. Dean, Andrew Rogers, Timothy W. Gant, Rachel Smith, Terry D. Tetley, Martin O. Leonard

**Affiliations:** 1 Toxicology Department, Centre for Radiation, Chemical and Environmental Hazards, Public Health England, Harwell Campus, Chilton, OX110RQ UK; 2 Environmental Hazards and Emergencies Department, Centre for Radiation, Chemical and Environmental Hazards, Public Health England, Chilton, Harwell Campus, Chilton, OX110RQ UK; 3 Lung Cell Biology, Airways Disease, National Heart & Lung Institute, Imperial College London, London, UK; 4 The National Institute for Health Research Health Protection Research Unit (NIHR HPRU), Health Impact of Environmental Hazards at King’s College London in partnership with Public Health England (PHE) in collaboration with Imperial College London, London, UK

**Keywords:** Asthma, Lung, Nanomaterial, Transcriptomics

## Abstract

**Background:**

Nanomaterial inhalation represents a potential hazard for respiratory conditions such as asthma. Cerium dioxide nanoparticles (CeO_2_NPs) have the ability to modify disease outcome but have not been investigated for their effect on models of asthma and inflammatory lung disease. The aim of this study was to examine the impact of CeO_2_NPs in a house dust mite (HDM) induced murine model of asthma.

**Results:**

Repeated intranasal instillation of CeO_2_NPs in the presence of HDM caused the induction of a type II inflammatory response, characterised by increased bronchoalveolar lavage eosinophils, mast cells, total plasma IgE and goblet cell metaplasia. This was accompanied by increases in IL-4, CCL11 and MCPT1 gene expression together with increases in the mucin and inflammatory regulators CLCA1 and SLC26A4. CLCA1 and SLC26A4 were also induced by CeO_2_NPs + HDM co-exposure in air liquid interface cultures of human primary bronchial epithelial cells. HDM induced airway hyperresponsiveness and airway remodelling in mice were not altered with CeO_2_NPs co-exposure. Repeated HMD instillations followed by a single exposure to CeO_2_NPs failed to produce changes in type II inflammatory endpoints but did result in alterations in the neutrophil marker CD177. Treatment of mice with CeO_2_NPs in the absence of HDM did not have any significant effects. RNA-SEQ was used to explore early effects 24 h after single treatment exposures. Changes in SAA3 expression paralleled increased neutrophil BAL levels, while no changes in eosinophil or lymphocyte levels were observed. HDM resulted in a strong induction of type I interferon and IRF3 dependent gene expression, which was inhibited with CeO_2_NPs co-exposure. Changes in the expression of genes including CCL20, CXCL10, NLRC5, IRF7 and CLEC10A suggest regulation of dendritic cells, macrophage functionality and IRF3 modulation as key early events in how CeO_2_NPs may guide pulmonary responses to HDM towards type II inflammation.

**Conclusions:**

CeO_2_NPs were observed to modulate the murine pulmonary response to house dust mite allergen exposure towards a type II inflammatory environment. As this type of response is present within asthmatic endotypes this finding may have implications for how occupational or incidental exposure to CeO_2_NPs should be considered for those susceptible to disease.

**Electronic supplementary material:**

The online version of this article (10.1186/s12989-018-0261-5) contains supplementary material, which is available to authorized users.

## Background

CeO_2_NPs have been used for many applications including precision polishing materials, oxide based fuel cells [1, 2] and as fuel catalysts [3]. Their unique redox properties have also led to investigation of their therapeutic potential for conditions where oxidative stress is indicated [4]. In the main, systemic or oral administration of CeO_2_NPs results in protection against injury in models of disease, including amyotrophic lateral sclerosis [5], Alzheimer’s disease [6], hepatic ischemic reperfusion injury [7] and drug induced cardiotoxicity [8]. On the other hand, exposure to CeO_2_NPs in the absence of underlying disease processes appears to result in toxicological effects, such as disruption of microvascular smooth muscle signalling [9], systemic organ toxicity [10] and potential genotoxicity [11, 12]. These observations of active biological interaction have led to concerns over whether CeO_2_NPs may pose a health hazard as a result of incidental or occupational exposure.

The potential effect on pulmonary health is a particular concern for inadvertent nanoparticulate exposure and CeO_2_NPs have been investigated in this context. Acute inhalation exposure of rats to CeO_2_NPs causes inflammatory effects, including neutrophil accumulation within the lung, an observation not seen when the CeO_2_NPs were coated with SiO_2_ [13]. Longer inhalation exposure over 1–4 weeks in rats was also found to induce neutrophil accumulation followed by macrophage dominated pulmonary inflammation. Interestingly, particle surface area rather than mass appeared to be a more appropriate metric of dose for predicting the pulmonary response [14]. However, when CeO_2_NPs were compared to larger micron sized CeO_2_ particles after 28 day inhalation, little quantitative differences in the pulmonary inflammation between materials was observed [15]. Sub-chronic exposures up to 90 days also resulted in inflammatory cell accumulation in the lung and were associated with impaired particle clearance [16]. While it is clear that CeO_2_NPs appear to have a detrimental effect on lung health in healthy animals, little information exists on how CeO_2_NPs may influence susceptible lung conditions such as COPD and asthma.

Asthma is an obstructive airway condition typified by airway hyperresponsiveness (AHR), bronchospasm and excessive mucus production [17]. Development of the condition as well as exacerbation events involve inflammatory processes, airway remodelling, smooth muscle effects and goblet cell metaplasia [18–21]. Experimental models have been used to assess the impact of nanomaterial exposure as a potential hazard for asthma and allergic airway disease [22]. These typically involve protocols of repeat exposure to allergens such as house dust mite (HDM) and assessment of inflammation, epithelial dysfunction and airway mechanics [23]. As the most common form of asthma is atopic in nature, models have focussed mainly on endpoints aimed at this endotype of disease [24]. These include increased levels of allergen specific IgE, mast cell activation and CD4+ T-cell patterning towards a type II (T_H_2) phenotype. These effects are associated with increases in IL-4, IL-5, IL-13 and CCL11 [25]. Type II inflammation involving innate lymphoid type 2 cells can also occur in the absence of allergic sensitisation and has been described as a contributing factor for other endotypes of disease such as intrinsic asthma [25, 26]. For both these conditions, injury to and activation of the airway epithelial cell layer is a prominent feature of type II mediated responses, with epithelial derived factors including CCL20, IL-33, TSLP, IL-25 and GM-CSF all playing a role in driving inflammatory responses. Phenotypical changes in these cells resulting in excessive mucin production, including mucin 5AC (MUC5AC), also support them as key contributors to the asthmatic phenotype [25].

In the absence of any toxicological information on how respiratory exposure to CeO_2_NPs may affect asthma, we set out to examine their effects using an HDM allergen exposure murine model of type II inflammatory lung disease [23]. Exposure to CeO_2_NPs and HDM was carried out across 9 separate intranasal exposures over 3 weeks and lung tissue was examined for alterations in inflammatory and mucin related changes as well as respiratory function. Alterations were observed with nanoparticle exposure and attempts were made to investigate the mechanisms involved through RNA-SEQ analysis of lung tissue after acute exposure. We also explored the translational nature of our in vivo observations and the role of the airway epithelium in more detail using in vitro exposures of air-liquid interface (ALI) cultures of the human bronchial airway epithelium.

## Results

### CeO_2_NP characterisation and deposition within the lung

**Fig. 1:**
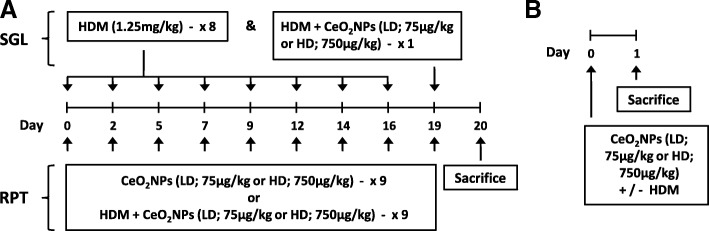
Experimental protocol for intranasal administration of HDM and nanoparticles. Balb/c mice were exposed through intranasal instillation to cerium dioxide nanoparticles (CeO_2_NPs), low dose (LD) (75 μg/kg) or high dose (HD) (750 μg/kg), alone or in combination with house dust mite (HDM) (1.25 mg protein/kg). Three week exposure protocols involved 9 individual instillations on the indicated days (**a**). Specific protocols involved either pre-treatment with HDM for the first 8 instillations followed by a combination of HDM + CeO_2_NPs (SGL) or 9 repeat treatments of CeO_2_NPs +/− HDM (RPT) as indicated (**a**). Mice were also exposed to a single instillation of CeO_2_NPs (LD and HD) +/− HDM (**b**). Sacrifice and collection of tissues was carried out on the days indicated (**a**, **b**)

**Fig. 2:**
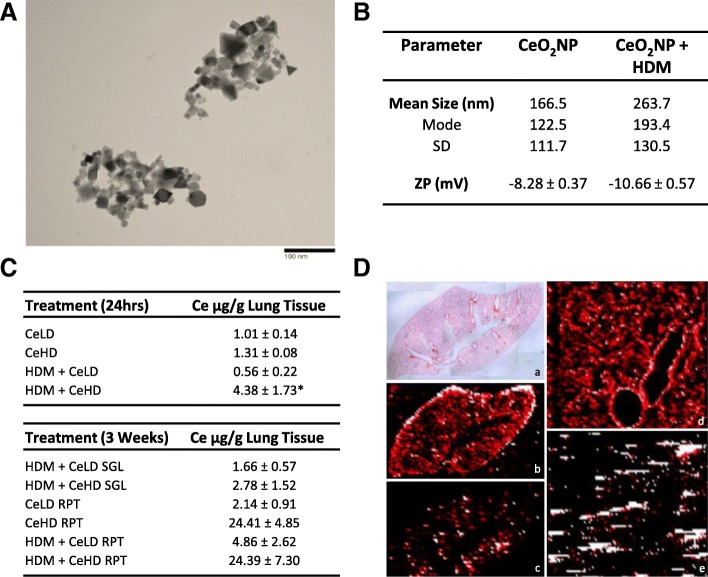
Nanomaterial characterisation and lung deposition. CeO_2_NPs were re-suspended in water, drops dried on TEM grids, and primary size and structure visualised using TEM (**a**). These nanoparticles were also suspended in PBS treatment diluent with and without HDM and agglomerate size was determined using nanoparticle tracking analysis (**b**) with results expressed as mean, mode and standard deviation (SD) of size distribution. Particle charge was also determined as zeta potential (ZP) by dynamic light scattering and expressed in millivolts (mV) mean values ± standard error of the mean (SEM) (**b**). Mice were exposed to CeO_2_NPs at either a low dose (CeLD) (75 μg/kg) or high dose (CeHD) (750 μg/kg) with and without HDM (1.25 mg/kg), instillation protocols as described in Fig. 1. After treatment, lung tissue was removed and digested prior to ICP-MS based quantification of elemental ^140^Ce content. Results are expressed as μg/g lung tissue mean values ± SEM for between 3 and 6 animals (**c**). Statistical significance between all treatments was carried out using one way ANOVA with comparison between CeHD and HDM + CeHD represented as (* *p* < 0.05). Lung tissue from a single exposure to CeHD for 24 h was fixed in 10% formalin and processed for laser ablation ICP-MS detection of ^63^Cu (b, d) and ^140^Ce (c, e) tissue distribution (**d**). The elemental distribution in both whole lung (a,b,c) (× 4 magnification) and airway (d,e) (× 100 magnification) was examined. H&E staining was used to visualise general lung structure (a) (**d**)

The ability of CeO_2_NPs to influence pulmonary responses to HDM allergens was investigated through a series of repeat and single exposure intranasal instillation protocols, as described in Fig. 1. Both repeat and single exposures of HDM and CeO_2_NPs, either alone or in combination, for up to 3 weeks were used. CeO_2_NPs used in this study have been reported by the manufacturer as having an average primary particle size of < 25 nm. TEM analysis of these particles (Fig. 2a) revealed primary particle size in line with this value. Multiple particle shape types were present. Agglomerate size was also determined in the PBS diluent used for exposure protocols and revealed a mean size of 166.5 nm (Fig. 2b). The mode and standard deviation (SD) values indicate a wide agglomerate size distribution. There was a modest increase in mean agglomerate size when HDM was added to the CeO_2_NPs. Surface charge was also assessed for CeO_2_NPs alone as a zeta potential of − 8.28 ± 0.37, which increased slightly when HDM was present. Ce elemental content within the lung was assessed using ICP-MS (Fig. 2c). At 24 h, Ce content within the lung after exposure to CeO_2_NPs at the lower dose (CeLD) (75 μg/kg) was not significantly different from the high dose (CeHD) (750 μg/kg). HDM (1.25 mg/kg) in combination with CeHD however resulted in a significant increase in Ce levels. At 3 weeks repeat exposure, Ce content within the lung was higher than at 24 h but was not significantly different between CeHD and HDM + CeHD. Ce content for both RPT CeHD treatments were increased above CeLD levels. No Ce was present within the lungs of PBS (CTRL) and HDM exposed mice for either 24 h or 3 week exposure protocols. Spatial distribution of Ce within the lung after instillation was also determined by laser ablation ICP-MS. We found disperse hotspots of cerium throughout the lung with occasional localised patterns around larger airways after 24 h (Fig. 2d) and repeat exposure protocols (data not shown). Spatial distribution was not observed to be different with HDM co-exposure.

### Repeat CeO_2_NP exposures modify the pulmonary inflammatory response to HDM

**Fig. 3:**
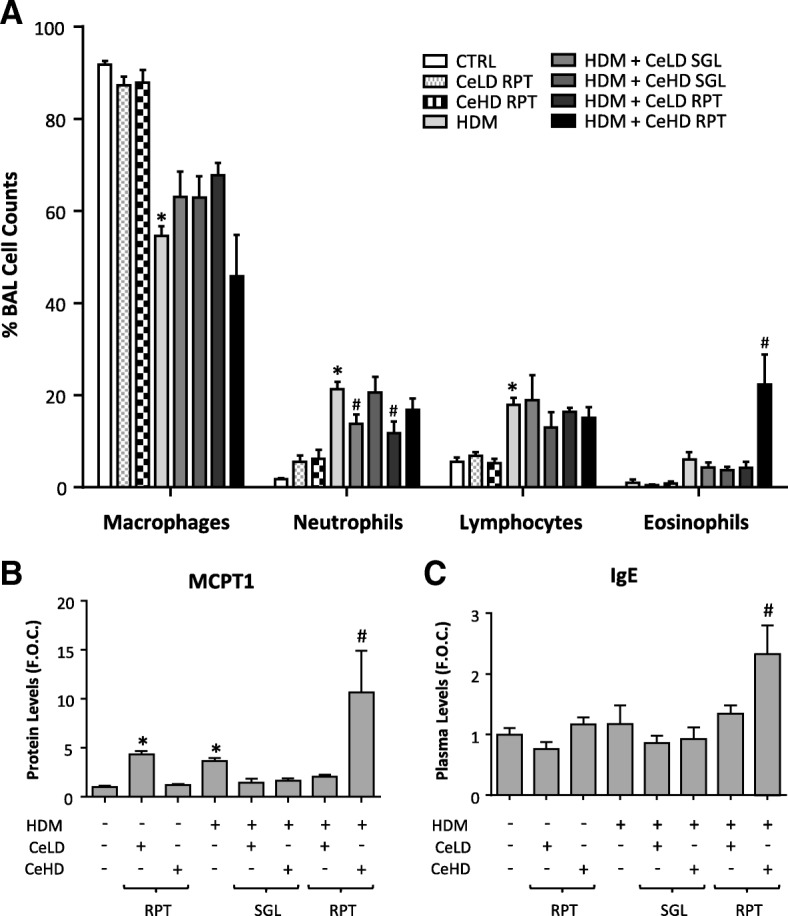
Inflammatory responses within the lung after repeat CeO_2_NPs and HDM exposure. Mice (*n* = 5–9 per treatment group) were exposed to CeO_2_NPs at either low dose (CeLD) (75 μg/kg) or high dose (CeHD) (750 μg/kg) with and without HDM (1.25 mg/kg), instillation protocols as described in Fig. 1. After treatment, bronchoalveolar cells were analysed for differential immune cell content. Results are expressed as mean ± SEM % total cells counted (300–500 per animal) (**a**). Lung homogenate was examined for protein levels of the mast cell marker MCPT1 (**b**) and total blood plasma Immunoglobulin E levels (**c**) by ELISA. Results were expressed as mean ± SEM fold over control (F.O.C.) levels. Statistical significance between treatments was carried out by one way ANOVA. Comparisons between particle and HDM treatments alone and control levels are represented as (* *p* < 0.05), while comparisons between particle + HDM combinations and HDM levels are represented as (# *p* < 0.05)

BAL cell analysis was carried out to profile the pulmonary inflammatory response to CeO_2_NP and HDM after repeat intranasal instillation exposure. Total BAL cell counts were increased with individual CeO_2_NP and HDM treatments (Additional file 1: Figure S1A). Co-exposure of CeO_2_NPs at the higher particle dose with HDM further increased total BAL cells above HDM induced levels both for SGL and RPT protocols. Differential BAL cell analysis was also assessed (Fig. 3a). HDM exposure caused an increase in neutrophil content, which was not altered by co-exposure with CeHD. It was however reduced by co-exposure to CeLD. Lymphocyte levels in the BAL were also increased by HDM but unmodified by CeO_2_NPs co-exposure. The percentage of eosinophils within the BAL was increased by HDM but not to a statistically significant level. This was not modified by CeLD co-exposure, but CeHD co-exposure resulted in a substantial increase in eosinophil levels for the RPT but not SGL exposure protocol (Fig. 3a). CeO_2_NP exposure in the absence of HDM did not result in any significant change in any BAL cell type. Mast cell content within the lung was also measured in lung homogenate using the marker MCPT1. Protein levels for this marker were induced by CeLD and HDM alone. HDM levels were further increased by co-exposure with CeHD only with the RPT protocol (Fig. 3b). Total blood plasma immunoglobulin E levels were also examined as an indicator of type II inflammation and revealed increased expression only with HDM + CeHD RPT protocol (Fig. 3c).

**Fig. 4:**
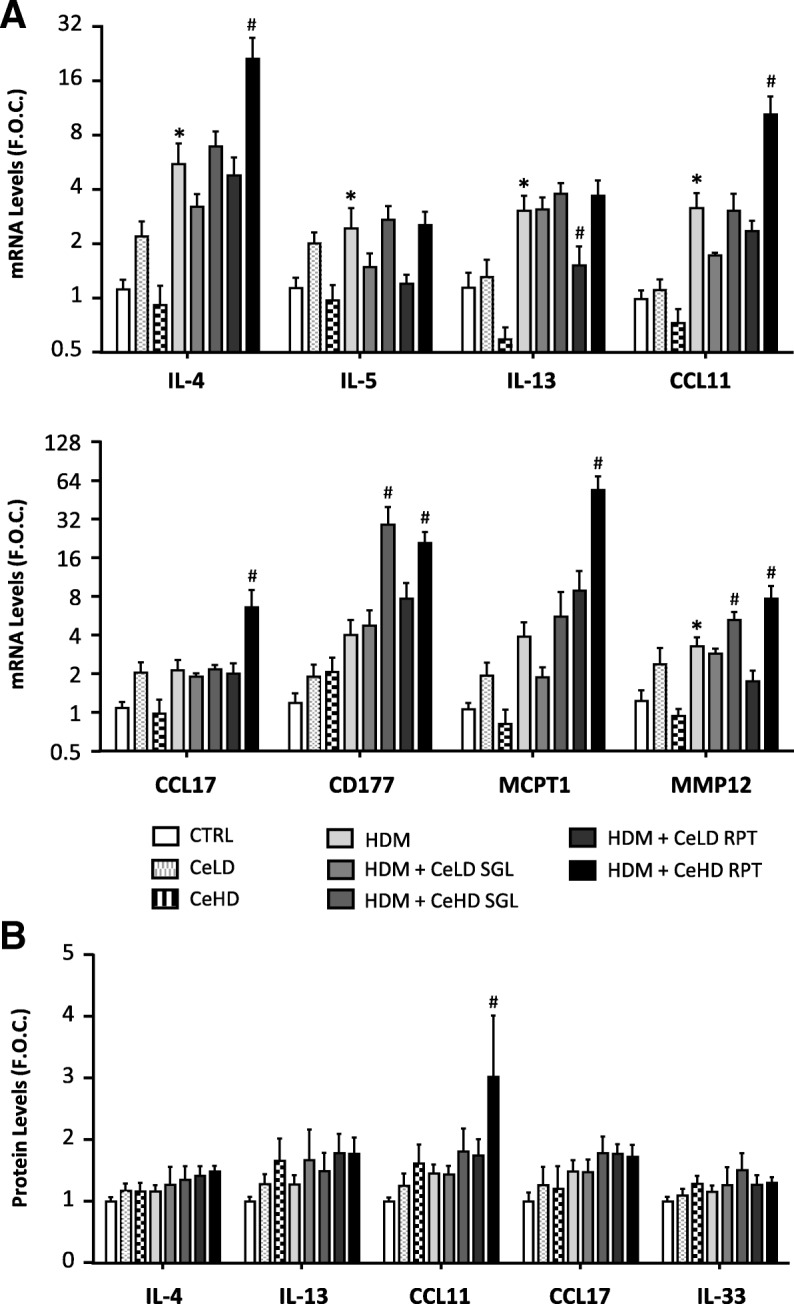
Inflammatory marker expression within the lung after repeat CeO_2_NPs and HDM exposure. Mice (*n* = 6–7 per treatment group) were exposed to CeO_2_NPs at either low dose (CeLD) (75 μg/kg) or high dose (CeHD) (750 μg/kg) with and without HDM (1.25 mg/kg), according to instillation protocols described in Fig. 1. After treatment, total lung mRNA was isolated and examined for transcript levels of the indicated inflammatory markers by RT-PCR analysis (**a**). BAL fluid was also examined for protein levels of inflammatory cytokines by ELISA (**b**). Results were expressed as mean ± SEM fold over control (F.O.C.) levels. Statistical significance between treatments was assessed using one way ANOVA. Comparisons between particle and HDM treatments alone and control levels are represented as (* *p* < 0.05), while comparisons between particle + HDM combinations and HDM levels are represented as (# *p* < 0.05)

The changes in cellular profiles indicated a shift towards type II inflammation. We therefore examined additional markers of this immune profile using analysis of lung tissue gene expression (Fig. 4a). HDM repeat exposure resulted in an increase in IL-4 and CCL11, which was further increased with CeHD RPT co-exposure. IL-5 and IL-13 were also increased with HDM treatment but CeO_2_NP exposure had limited effects on these levels. CCL17 and MCPT1 mRNA levels were significantly upregulated only by HDM + CeHD RPT exposure. Protein levels for select type II inflammatory mediators were also examined in BAL fluid (Fig. 4b). Neither HDM nor CeO_2_NPs alone resulted in any significant change in protein expression. There was however an increase in CCL11 with HDM + CeHD RPT exposure compared to HDM alone (Fig. 4b). CeO_2_NP exposure in the absence of HDM did not result in any significant change in any marker expression. A summary of the impact of different treatment regimens on pulmonary inflammation is provided in Table 1.

**Table 1:** Summary effect of HDM and CeO_2_NP co-exposure on pulmonary inflammation and airway dysfunction

	*Bronchoalveolar lavage cells*				*Lung inflammatory indicators mRNA (protein)*									*Airway mucin, structure and mechanics*							
	Total	Neu	Lym	Eos	MCPT1	IL-4	IL-5	IL-13	CCL11	CCL17	CD177	IL33	*Plasma IgE*	Mucin	Clca1	Muc5ac	SLC26A4	M.T.	α-sma	PCNA	AHR (Rrs)
CeLD RPT	↑	nc	nc	nc	nc (↑)	nc (nc)	nc	nc (nc)	nc (nc)	nc (nc)	nc	(nc)	nc	nc	nc	nc	nc	nc	nc	nc	nc
CeHD RPT	↑	nc	nc	nc	nc (nc)	nc (nc)	nc	nc (nc)	nc (nc)	nc (nc)	nc	(nc)	nc	nc	nc	nc	nc	nc	nc	nc	nc
HDM	↑	↑	↑	nc	nc (↑)	↑ (nc)	↑	↑ (nc)	↑ (nc)	nc (nc)	nc	(nc)	nc	↑	nc	nc	nc	nc	↑	↑	↑
HDM + CeLD SGL	nc	↓	nc	nc	nc (nc)	nc (nc)	nc	nc (nc)	nc (nc)	nc (nc)	nc	(nc)	nc	nc	nc	nc	nc	nc	nc	nc	nc
HDM + CeHD SGL	↑	nc	nc	nc	nc (nc)	nc (nc)	nc	nc (nc)	nc (nc)	nc (nc)	↑	(nc)	nc	nc	↑	↑	nc	nc	nc	nc	nc
HDM + CeLD RPT	nc	↓	nc	nc	nc (nc)	nc (nc)	nc	↓ (nc)	nc (nc)	nc (nc)	nc	(nc)	nc	nc	nc	nc	nc	nc	nc	nc	nc
HDM + CeHD RPT	↑	nc	nc	↑	↑ (↑)	↑ (nc)	nc	nc (nc)	↑ (↑)	↑ (nc)	↑	(nc)	↑	↑	↑	↑	↑	nc	nc	nc	nc

Pulmonary and systemic responses to intranasal administration of HDM and CeO2NP treatment over 3- week administration regimens is provided as a qualitative metric for cross treatment and protocol comparison purposes. Treatments are indicated within the first column with protocol details as described in Fig. 1. For CeLD RPT, CeHD RPT and HDM treatments, comparison against control animals is represented as an increase (↑), a decrease (↓) or no change (nc) for each measurement. For HDM + CeLD SGL, HDM + CeHD SGL, HDM + CeLD RPT and HDM + CeHD RPT treatments, comparison against HDM treated animals is represented as an increase (↑), a decrease (↓) or no change (nc) for each measurement. Abbreviations used: Neu, neutrophils; Lym, lymphocytes; Eos, eosinophils; M.T., Masson’s Trichrome; α-sma, alpha smooth muscle actin; AHR, airway hyperresponsiveness; Rrs, Resistance

### Effect of repeat CeO_2_NP and HDM exposures on airway remodelling and respiratory function

**Fig. 5:**
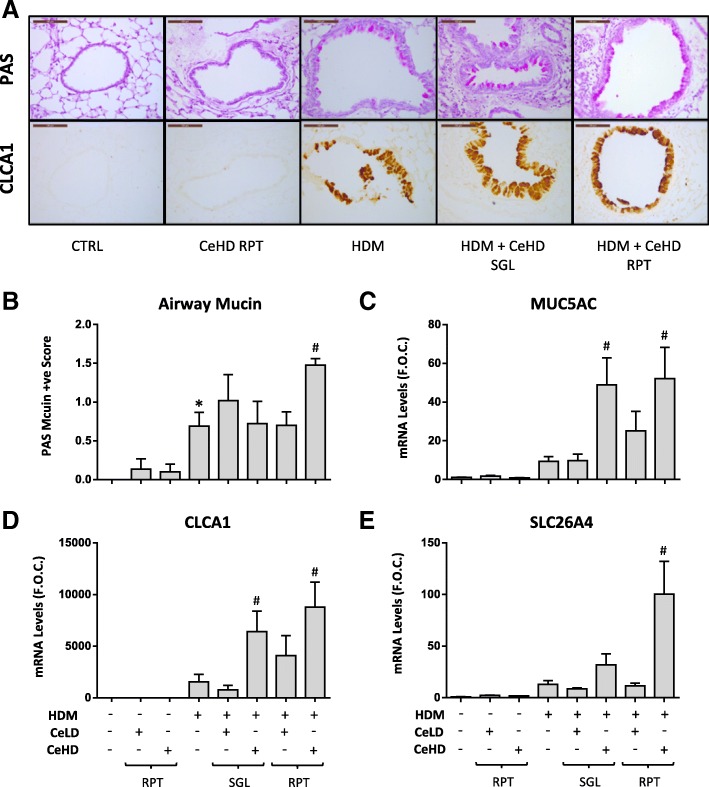
CeO_2_NPs and HDM alter airway mucin and marker expression after repeat exposure. Mice (n = 6–7 per treatment group) were exposed to CeO_2_NPs at either low dose (CeLD) (75 μg/kg) or high dose (CeHD) (750 μg/kg) with and without HDM (1.25 mg/kg), according to instillation protocols described in Fig. 1. Lung tissue was fixed and sections processed for PAS staining (**a**; Top panels) or immunohistochemical detection of CLCA1 protein (**a**; Bottom panels). Representative images are displayed with a scale bar of 100 μm. Quantification of airway mucin positive cells was also carried out and displayed as mean ± SEM PAS score (**b**). Total lung mRNA was also examined for mucin related gene expression by RT-PCR analysis (C-E) and results expressed as mean ± SEM fold over control (F.O.C.) levels. Statistical significance between treatments was assessed using one way ANOVA. Comparisons between particle and HDM treatments alone and control levels are represented as (* *p* < 0.05), while comparisons between particle + HDM combinations and HDM levels are represented as (# *p* < 0.05)

Goblet cell metaplasia with increased mucin production is one of the hallmark indicators of allergic airway inflammation [27]. PAS histochemical staining can be used to characterise airway structure to determine the extent to which the airway epithelium produces excessive mucin and to determine the extent of goblet cell metaplasia. We identified mucin positive cells in airways between 100 and 250 μm diameter (Fig. 5a; Top panels), and quantified (Fig. 5b). HDM was observed to induce mucin positive cells above CTRL levels, which was further increased by co-exposure to RPT CeHD. MUC5AC is considered a major component of airway mucin and mRNA levels were observed to increase with HDM but not to a significant level (Fig. 5c). HDM + CeHD RPT and SGL treatments caused a significant increase in MUC5AC when compared to HDM alone. CLCA1, which has been closely associated with the regulation of airway mucin [28], was also examined. HDM induced an increase in airway protein levels for CLCA1, which, similar to mucin expression, was exclusively restricted to the airway epithelium of larger airways (Fig. 5a; Bottom panels). CeHD co-exposure with HDM both with RPT and SGL protocol treatments appeared to further increase CLCA1 expression within the airways. Similar effects were observed at the mRNA level for CLCA1, where CeHD co-exposure also significantly increased levels above HDM alone (Fig. 5d). No staining for CLCA1 in lung tissue and very low mRNA levels were observed for CTRL or CeHD RPT alone exposures. SLC26A4 is another bronchial marker protein expressed in asthma and COPD, which has been suggested to contribute to mucin regulation [29]. Expression levels for SLC26A4 were also found to be significantly upregulated by HDM + CeHD RPT when compared to HDM levels alone (Fig. 5e).

**Fig. 6:**
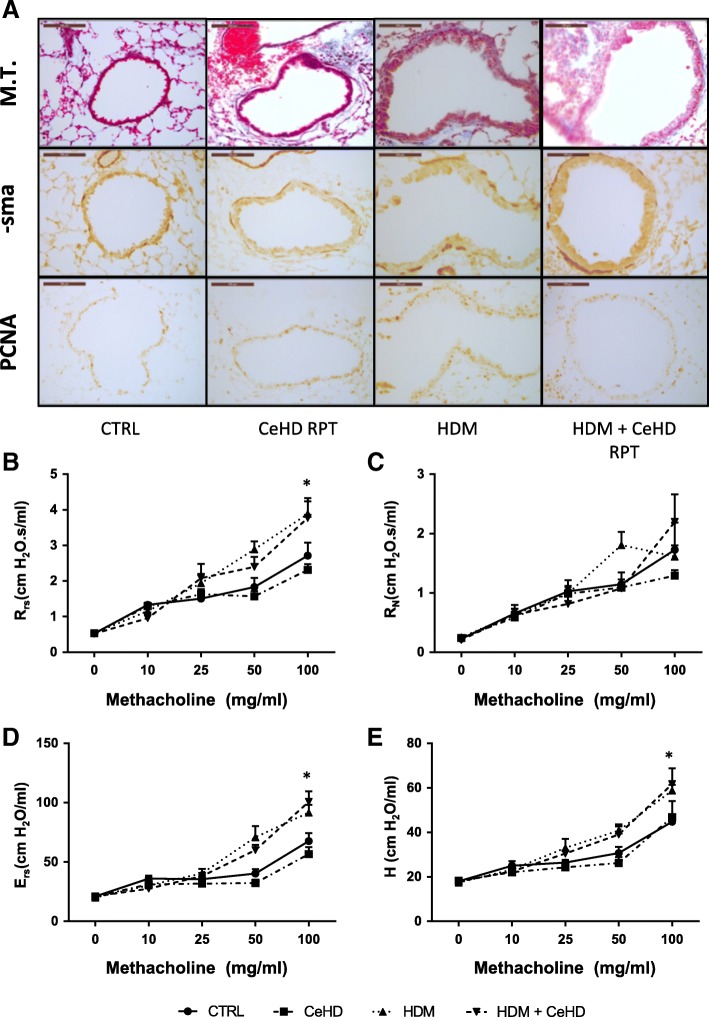
Airway structure and lung function assessment after repeat CeO_2_NPs and HDM exposure. Mice (*n* = 6–7 per treatment group) were exposed to CeO_2_NPs at the higher dose (CeHD) (750 μg/kg) with and without HDM (1.25 mg/kg) 9 times over a period of 3 weeks. Lung tissue was fixed and sections processed for Masson’s Trichrome (M.T.) staining (**a**; Top panels) and immunohistochemical detection of α-sma (**a**; Middle panels) or PCNA (**a**; Bottom panels). Representative images are displayed with a scale bar of 100 μm. Mice were also assessed for airway hyperresponsiveness to increasing concentrations of inhaled methacholine aerosol (**b**-**d**) using the forced oscillation technique. Respiratory system resistance (Rrs) (**b**), elastance (Ers) (**d**) as well as Newtonian resistance (R_N_) (**c**) and tissue elastance (H) (**e**) were calculated within flexivent system software and expressed as mean + SEM cmH_2_O. Statistical significance between treatments at each concentration of methacholine was carried out using student t-test (* *p* < 0.05) compared to control group

Airway remodelling is a critical component of asthma pathogenesis, underlying airway hyperresponsiveness. Masson’s trichrome staining was used to assess peribronchial collagen levels (Fig. 6a; Top panels) and did not reveal any significant difference with treatment. Alpha smooth muscle actin, as a surrogate marker for airway smooth muscle content was also assessed and was demonstrated to have increased levels with HDM RPT treatment, which was not modified by CeHD co-exposure (Fig. 6a; Middle panels). PCNA staining was also examined to assess proliferation, which can accompany airway remodelling (Fig. 6a; Bottom panels). HDM increased PCNA positive cells but CeHD co-exposure did not appear to modify this effect. Respiratory mechanics were also examined as airway responsiveness to increasing concentrations of methacholine. HDM repeat treatment was observed to increase respiratory system resistance and elastance as well as tissue elastance, which was not further modified with co-exposure to CeHD (Fig. 6b-e). A summary of the impact of the different treatment regimens on airway remodelling and respiratory function is provided in Table 1.

### Short term effects of airway exposure to CeO_2_NP and HDM

**Fig. 7:**
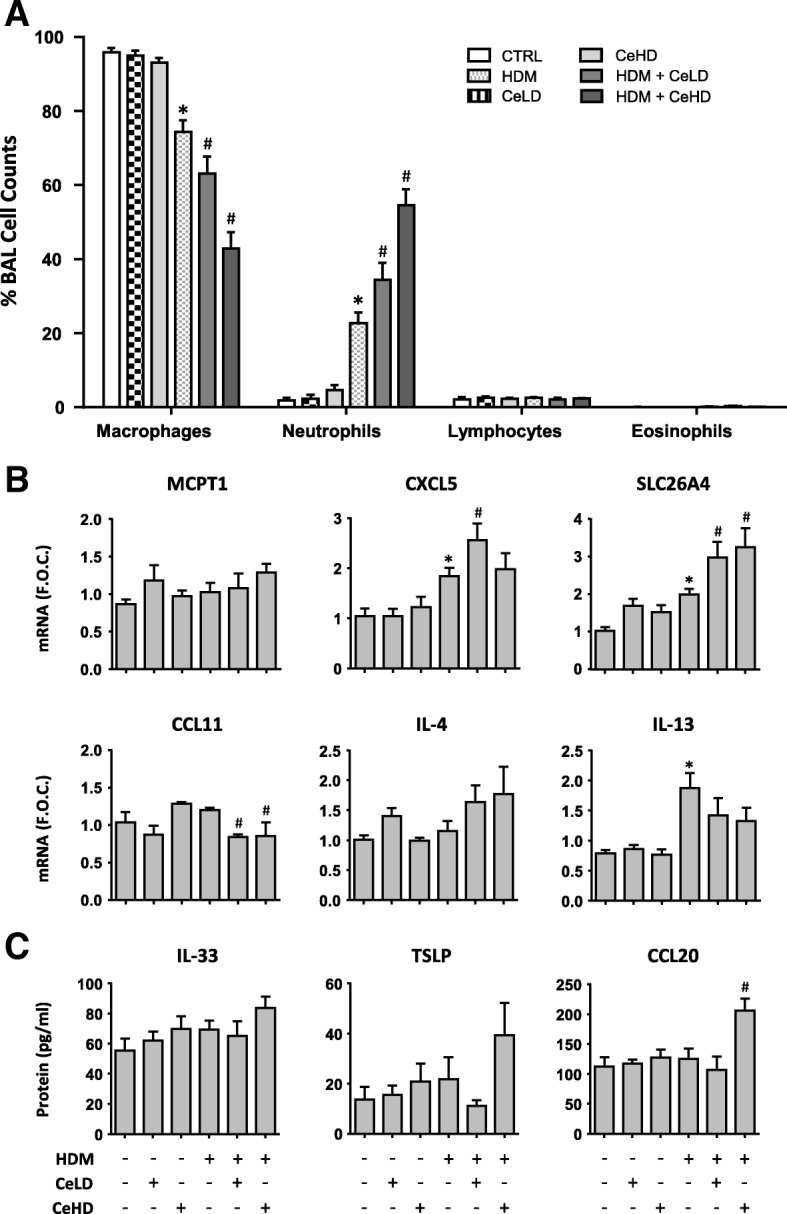
Inflammatory responses within the lung after single CeO_2_NPs and HDM exposure. Mice (*n* = 6 per treatment group) were exposed to CeO_2_NPs at either low dose (CeLD) (75 μg/kg) or high dose (CeHD) (750 μg/kg) with and without HDM (1.25 mg/kg) for 24 h. After treatment, bronchoalveolar cells were analysed for differential immune cell content. Results are expressed as mean ± SEM % total cells counted (300–500 per animal) (**a**). After treatment, total lung mRNA was isolated and examined for transcript levels of the indicated inflammatory markers by RT-PCR analysis with results expressed as mean ± SEM fold over control (F.O.C.) levels (**b**). BAL fluid was also examined for protein levels of inflammatory cytokines by ELISA (**c**) and results expressed as mean ± SEM pg/ml. Statistical significance between treatments was assessed using one way ANOVA. Comparisons between particle and HDM treatments alone and control levels are represented as (* *p* < 0.05), while comparisons between particle + HDM combinations and HDM levels are represented as (# *p* < 0.05)

To further our understanding of the principal mechanisms involved in the modifying effects of CeO_2_NPs in this HDM asthma model, we carried out a series of single instillation exposures for 24 h. Total BAL cell counts were increased with single CeO_2_NP and HDM treatments alone (Additional file 1: Figure S1B). Co-exposure of CeO_2_NPs at the higher dose with HDM further increased total cells above HDM induced levels. HDM exposure caused an increase in BAL neutrophil counts, which was further enhanced by both CeLD and CeHD co-exposure (Fig. 7a). CeLD or CeHD alone did not have any effect on BAL cell populations. No effects of any treatment were observed for lymphocyte or eosinophil content within the BAL (Fig. 7a) nor for the mast cell marker MCPT1 (Fig. 7b). Gene expression levels for the neutrophil chemoattractant CXCL5 were increased for HDM and further increased with CeLD co-exposure (Fig. 7b). SLC26A4 mRNA levels were increased with HDM and further increased with CeO_2_NP co-exposure. Levels of expression for CCL11 indicated an inhibitory effect for CeO_2_NP co-exposure, while IL-4 and IL-13 levels were not modified. The initiation of type II inflammatory effects and allergic sensitisation in the airways involves the innate immune system including epithelial derived IL-33, TSLP and CCL20, which act to attract and activate dendritic cells. Protein levels of these mediators were examined (Fig. 7c). No statistically different effects were observed for IL-33 and TSLP, although there would appear to be some indication that co-exposure of HDM with CeHD may produce some level of increase. Expression levels however for CCL20 were significantly upregulated for HDM + CeHD as compared to HDM alone (Fig. 7c). IL-1α and IL-4 protein levels in the BAL were also assessed but did not result in any significant alterations (data not shown). PAS staining of lung tissue did not reveal any indication of mucin positive cells within the airways after 24 h exposure (data not shown).

**Fig. 8:**
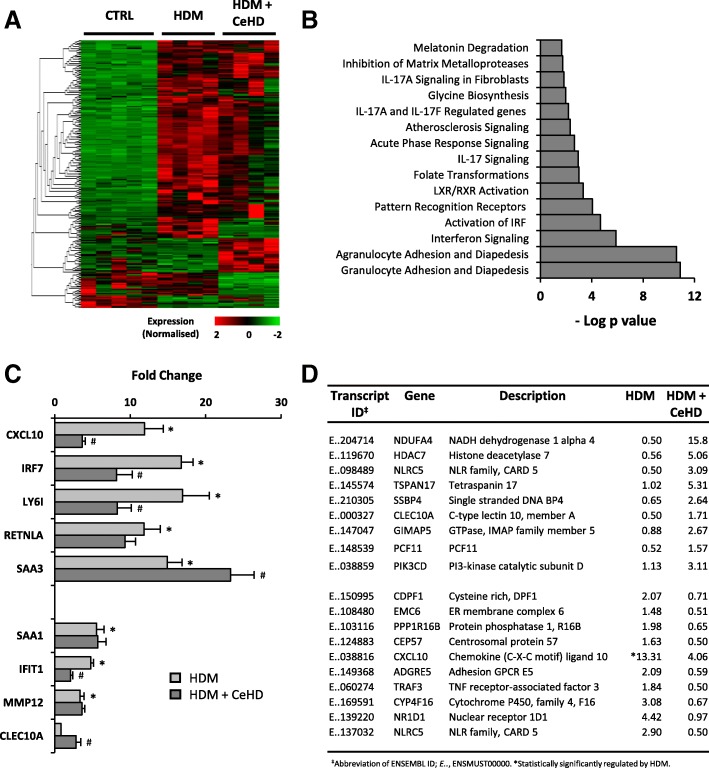
RNA-Seq analysis after single CeO_2_NPs and HDM exposure. Mice (*n* = 5 per treatment group) were exposed to HDM (1.25 mg/kg) alone or in combination with CeO_2_NPs at the higher dose (CeHD) (750 μg/kg) for 24 h. Total lung mRNA was isolated and Truseq library prepared prior to 90PE sequencing analysis. RPKM normalised counts were analysed for statistically differentially regulated transcripts between all exposure groups using Qlucore software (*p* < 0.005) using a 2 fold cut-off. Significantly regulated transcripts were visualised as a heatmap of normalised RPKM values (**a**). Regulated transcripts induced by HDM were analysed for pathway association using IPA analysis with results displayed as statistically ranked associations (**b**). Selected transcript expression is shown as mean ± SEM fold over control RPKM values (**c**). Statistical significance between treatments was assessed using one way ANOVA. Comparisons between control (CTRL) and HDM treatments are represented as (* *p* < 0.05), while HDM vs HDM + CeHD are represented as (# *p* < 0.05). The most highly regulated transcripts by HDM + CeHD over HDM levels are displayed (**d**)

Global gene expression alterations for HDM and HDM + CeHD were also examined using RNA-SEQ (Fig. 8). A heatmap representation of those differentially regulated transcripts is depicted (Fig. 8a) and demonstrates the majority of HDM induced changes to be upregulated. CeHD + HDM also resulted in differential transcript expression independent of HDM regulation. Pathway analysis of those transcripts regulated by HDM alone identified innate immune responses, including granulocyte processes and interferon signalling, as the most prominent (Fig. 8b). Expression of a selection of genes representative of these pathways and within the top 10 highest regulated genes by HDM, for both HDM and HDM + CeHD are displayed (Fig. 8c). These include CXCL10, IRF7, LY6I and IFIT1, which are upregulated by HDM. Pathway analysis also revealed that IRF3 and IRF7 as the most significant transcriptional regulators responsible for driving HDM induced gene expression (data not shown). This level of expression was modified by co-exposure with CeHD to levels significantly lower than HDM alone. Interestingly CeHD was observed to increase expression of SAA3 and CLEC10A above HDM regulated levels. A full list of the most highly regulated transcripts both up and downregulated are displayed in Fig. 8d.

Evidence thus far would indicate a role for the airway epithelium as a central player in the modifying effect of CeO_2_NP co-exposure on the HDM response within the lung. To determine whether such observations can be translated to a representative human model, we exposed ALI differentiated cultures of human primary bronchial epithelial cells to HDM and CeO_2_NPs. Transcript expression levels of epithelial derived inflammatory and mucin related signals were examined and summarised as a heatmap (Additional file 1: Figure S2A). HDM alone produced little effect on gene expression. For the majority of responses to CeO_2_NP co-exposure, repeat apical treatments over 1 week produced more significant differences than single exposure for 24 h. Select genes were visualised in greater detail (Additional file 1: Figure S2B). The neutrophil chemoattractant IL-8 displayed significantly higher levels of expression with HDM + ce1340 as compared to HDM alone for 1 week exposure. Similarly, the mucin related genes SLC26A4 and CLCA1 also displayed higher levels of expression with the higher doses of CeO_2_NPs at 1 week but not after single exposure. There were no observable alterations in toxicity or cellular dysfunction with any of the treatments, determined as release of LDH and lactate levels into the apical and basolateral compartments (data not shown).

## Discussion

Repeat exposure to house dust mite allergens over 3 weeks resulted in pulmonary inflammation, characterised as increases in neutrophils, lymphocytes and mast cells. CeO_2_NP co-exposure at the highest dose resulted in eosinophilia, increased plasma IgE as well as enhanced mast cell marker expression. HDM induced increases in the inflammatory cytokines IL-4 and CCL11, which are responsible for B cell class switching to IgE production and eosinophil recruitment respectively [25], were further increased by CeO_2_NP exposure. In addition, goblet cell metaplasia and MUC5AC expression were significantly induced. These observations are consistent with the induction of type II inflammation. The mechanisms involved in driving this type of inflammation could not be specifically attributed, but may involve adaptive immune T_H_2 T-cells or non-atopic mechanisms involving ILC2 cells [25, 30]. Lymphocyte levels within the BAL were induced by HDM but remained unaltered by CeO_2_NP co-exposure. This does not exclude the possibility of different profiles of lymphocytes with CeO_2_NP treatment. Mast cells and basophils can produce mediators and drivers of type II inflammation such as IL-4 [25, 31]. Interestingly, mast cells have been previously observed to control inflammatory processes within the lung in response to CeO_2_NP exposure [32] strengthening the possibility that CeO_2_NPs exert part of their influence on type II inflammation in our study through mast cell recruitment and activation. CeO_2_NP co-exposures were not observed to influence airway hyperresponsiveness over changes induced by HDM alone. As our model design aimed to capture acute responses, it is interesting to speculate whether longer term exposure to CeO_2_NPs and HDM together with a persistent type II inflammatory environment would influence airway remodelling and respiratory mechanics.

Pathological alterations in the conducting airway compartment drive asthmatic disease and therefore were the main focus for our study. When compared to inhalation exposure, pulmonary instillation techniques result in greater deposition of material in the bronchial/bronchiolar regions as compared to more distal parts of the lung [33]. Studies have shown that intranasal instillation of a 25 μl volume typically results in approximately 50% deposition of material within the lung [34]. To examine deposition patterns within the lung, we used LA-ICP-MS. 24 h after instillation; cerium was widely distributed throughout the lung with localised hotspots likely representing clearance activity by phagocytes. Given the relatively rapid clearance of particles from the mouse conducting airways [35], cerium levels at times before the 24 h time point would likely reveal widespread conducting airway coverage, before phagocytotic clearance and material redistribution. Interestingly, Ce levels in the presence of HDM were higher than with CeO_2_NP treatment alone after 24 h exposure. It could be suggested that the presence of HDM resulted in a slower clearance as a mechanism for increased nanoparticle retention and increased biological impact. However since this effect was not observed with repeat treatments, the contribution to enhanced biological effects is uncertain. The behaviour of CeO_2_NPs in the presence of HDM may also be different. CeO_2_NPs + HDM produced a larger agglomerate size and altered zeta potential. These were however within the error range and therefore unlikely to explain differences in biological effect. A more likely explanation stems from the ICP-MS measurements. When compared to 24 h single exposure, repeat treatment with CeO_2_NPs, either in the presence or absence of HDM resulted, in a substantially greater quantity of Ce within the lung, especially for the CeHD. As no inflammatory responses were observed with repeat exposure to CeO_2_NPs in the absence of HDM, we can suggest that a higher dose of CeO_2_NPs modifies HDM effects towards type II inflammation. This is also supported by the observations that a single CeO_2_NP exposure given after repeated HDM exposure failed to induce type II inflammation, although HDM did appear to prime for some responses including neutrophil effects.

BAL cell analysis 24 h after a single exposure to HDM resulted in significant neutrophil accumulation, which was further enhanced with CeO_2_NPs co-exposure. Levels of SAA3, which strongly correlates to neutrophil influx into pulmonary tissue in response to nanoparticle exposure [36], followed the pattern of BAL neutrophil levels with HDM and CeO_2_NPs exposures. As serum amyloid A acts as a neutrophil chemoattractant and is released from macrophages [37, 38] we suggest that given the spatial distribution of cerium in lung tissue after 24 h, indicative of phagocytosis, that macrophages are a likely early responder and influence subsequent tissue responses including neutrophil recruitment. CeO_2_NPs alone did not induce neutrophil recruitment, with increases in total BAL cells at both 24 h and repeat exposures likely due to increased macrophage content. Neutrophils are active phagocytes and have the potential to ingest particles including nanomaterials to influence their function [39, 40]. They have also been observed to induce type II inflammation through the release of neutrophil extracellular (NET) traps containing genomic DNA, in a mouse model of rhinovirus induced asthma exacerbation [41]. Markers specific to neutrophils include CD177, which when expressed has roles in endothelial migration [42] as well as reactive oxygen species (ROS) production and NET formation [43]. It is interesting to speculate whether the CeO_2_NP induced increases in CD177 expression in lung tissue in HDM primed SGL and co-exposed RPT protocols play a role in the induction of type II inflammation, and whether this may involve the regulation of NET formation.

The primary mechanisms by which CeO_2_NPs modulate type II inflammation were further investigated. RNA-Seq and pathway analysis of exposure to HDM for 24 h revealed a profile of gene expression within the lung predominantly of granulocyte recruitment to inflamed tissues and fits with our observations of neutrophil accumulation within the lung. Moreover, there was also a significant induction of genes associated with type I interferon signalling including IRF7, OAS1, IRF9, IFIT3, ISG15 and IFITM3. Prediction software to identify transcriptional regulators responsible for HDM induced gene expression revealed IRF7 and IRF3 as the most significant regulators. Whether IRF3 and the control of interferon type I signalling and related gene expression is involved in the promotion of allergic airway inflammation in response HDM is somewhat unclear. Evidence however, would suggest that gene expression changes in our study support a role for IRF3 and regulation of type I interferon related genes in the early responses to HDM and CeO_2_NPs. For example, CeO_2_NP co-exposure with HDM inhibited the expression of OAS1G, MX1, MX2, NLRC5, RTP4, LY6I, IRF7, CXCL10, OASL1, OAS3 and CXCL9, all of which were observed as IRF3 dependent in lung DCs [44]. How these early changes may translate to regulation of type II inflammatory effects is unknown but one could consider modulation of dendritic cells and the balance of T-cell differentiation as a component part. Indeed, the involvement of DC recruitment in the responses to HDM and CeO_2_NPs co-exposure are indicated from the increased CCL20 protein expression within the lung after 24 h single exposure.

Besides DC and T-cells, other immune cells are likely to play a major role in controlling the type of inflammatory response to CeO_2_NPs. CeO_2_NP co-exposure with HDM after 24 h resulted in the induction of CLEC10A. This gene is a known marker of M2 macrophage polarisation [45, 46], an important macrophage immunological profile responsible for driving anti-inflammatory/repair processes as well as promoting type II inflammatory tissue responses through the M2a sub-phenotype [40], including allergic airway responses within the asthmatic lung [47]. RETNLA another marker of M2 macrophage polarisation was strongly induced by HDM but unaltered by CeO_2_NP co-exposure. Interestingly, CeO_2_NPs have been documented to cause a shift in macrophage phenotype in the rat lung towards M2 [48, 49] and this has been suggested as a mechanism through which they promote pro-fibrotic activity within the lung. Polarisation of macrophages towards the M2 phenotype early on in HDM and CeO_2_NP co-exposure is therefore likely, but the precise nature and subcategory of M2 profile, as well as the precise role in promoting type II inflammation requires further investigation.

It has been suggested that the unique ability of CeO_2_NPs to modify oxidative potential underlies their ability to influence biological processes after cellular exposure [50]. We have previously reported that CeO_2_NPs can reduce oxidative stress induced alterations in epithelial injury and transcriptional responses [51]. It has also been reported that the ability of CeO_2_NPs to influence DC function towards modulation of T-cells to a T_H_2 phenotype was related to their ability to reduce ROS induced inflammasome activation [52]. We investigated whether markers of oxidative stress were altered with HDM and CeO_2_NP treatment by PCR analysis of mRNA and found no changes in HMOX1 or NQO1 with any exposure protocol used in this study. It has been demonstrated that HDM repeat pulmonary exposure can produce oxidative damage and DNA double strand breaks in the lungs and is suggested as the driver of asthma pathophysiology [53]. Whether CeO_2_NPs are modulating HDM induced oxidative stress and inhibiting Th1 promoting ability of HDM, thus allowing predominant T_H_2 responses and type II inflammatory environments to prevail [52] is untested but consistent with observations in our study.

In addition to immune cell activation as a key mediator of CeO_2_NPs effects, the epithelial response is also important. HDM has been observed to mediate allergic airway disease through epithelial expression and activation of the PRR TLR4, and downstream DC activation as a consequence of endotoxin present within the HDM [54]. TLR4 mediated airway effects have also been observed to be activated through HDM-induced TLR activation via dual oxidase 2-mediated reactive oxygen species [55] and may represent a target for redox modulating capability of CeO_2_NPs. Epithelial specific responses within lung tissue on repeat treatments were observed as increases in airway mucin, MUC5AC and the regulators CLCA1 and SLC26A4. Interestingly these later two proteins have been associated with asthmatic disease and are regulated by type II inflammatory mediators such as IL-13 [28, 56, 57]. The significance of these alterations and the role of the airway epithelium in directing tissue responses were also supported by increased expression of these genes in human bronchial airway epithelial ALI cultures, which also included regulation of CXCL5. Our experimental approach using ALI cultures did not allow for direct aerosol particle exposure but rather application of a nanoparticle suspension to the apical compartment. While we acknowledge differences may arise due to the method of particle application, we believe our observations to be significant in our attempts to develop translational in vitro model testing strategies for pulmonary exposure.

Finally, it is a priority within nanotoxicology research to identify those nanomaterial properties that can be associated with adverse outcome. Toxicological testing to date using models of asthma and AAD, has attempted this but difficulties surrounding diverse model parameters, dosing regimen, exposure types as well as the lack of material characterisation across studies makes extrapolation of reliable hazard information difficult [22]. Apart from the CeO_2_NPs used in our current study, testing for nanomaterial effects on HDM induced pulmonary outcomes has only been carried out for carbon nanotubes. MWCNT intranasal co-administration with HDM produced similar changes in immune cells, select cytokine expression and mucin production when compared to CeO_2_NPs used in our study. There did appear to be however greater tissue remodelling and fibrotic changes with MWCNT exposure [58]. Interestingly, the fibrotic effect of MWCNT has also been observed in another model of HDM induced airway inflammation [59]. Model parameter differences preclude direct comparison to CeO_2_NP effects in our study but it is interesting to speculate whether properties inherent to MWCNTs such as a fibre-like structure may contribute to fibrotic pathology on pulmonary exposure.

## Conclusion

CeO_2_NPs were observed to modulate the murine pulmonary response to HDM allergen exposure towards a type II inflammatory environment. This type of response is present within atopic and non-atopic asthmatic endotypes and may have implications for how occupational or incidental exposure to CeO_2_NPs should be considered for those susceptible to disease. Through mechanistic investigation we suggest that CeO_2_NPs modulate specific interferon signalling events including IRF3 to direct inflammatory responses. Whether such responses are under the control of the redox modifying capabilities of cerium dioxide is unknown but warrants further investigation.

## Methods

### Nanoparticle preparation and characterisation

CeO_2_NPs were obtained from Sigma Aldrich (Dorset, United Kingdom) (cat # 544841). Particles were suspended in distilled H_2_O before sonication (QSonica Sonicators, CT, USA) with 4.2x10^5^kJ/m^3^. For transmission electron microscopy (TEM), particles were placed onto Formvar/Carbon 200 Mesh Copper TEM grids and visualized using a JEOL 3000F instrument (JEOL Inc., Tokyo, Japan). CeO_2_NPs were also suspended in phosphate buffered saline (PBS) +/− HDM (Greer Laboratories, Lenoir, cat# XPB82D3A25) prior to size and charge characterisation. The particle size distribution was determined by nanoparticle tracking analysis using a NanoSight LM10 instrument (NanoSight, Amesbury, UK). Instrument recordings were at least 60 s and processed using NTA 3.2 Analytical software. Particle charge was determined as zeta potential by dynamic light scattering using a Zetasizer Nano (Malvern, Malvern United Kingdom). Size and zeta potential measurements were carried out for 3 technical replicates across 3 separate samples.

### In vivo exposure and tissue collection

Female Balb/c mice between 6 and 8 weeks (Envigo, UK) were anaesthetised with 5% isoflurane in oxygen using a precision vaporizer and intranasally instilled with 25 μl of combinations of CeO_2_NPs with and without HDM in different combinations and at different intervals, as described in Fig. 1. Instillation of an equivalent dosing volume of PBS was carried out as the control procedure. After the treatment period, mice were euthanized using an overdose of 0.1 mL sodium pentobarbital (200 μg/mL) by intraperitoneal injection and exsanguination by cardiac puncture. Blood was collected into EDTA coated tubes and plasma was isolated by centrifugation at 1500×g for 15 min at 4 °C. Lung tissue was removed after bronchoalveolar lavage (BAL), washed in sterile PBS and either frozen for later analysis or perfused with 10% formaldehyde solution.

### BAL cell analysis

Immediately after sacrifice BAL fluid was collected for assessment of the inflammatory responses in the lungs. Briefly, the trachea was exposed and cannulated allowing the lungs to be lavaged with 500 μl ice-cold sterile PBS. The lungs were gently aspirated and the process repeated a further two times (1.5 ml total). BAL fluid was centrifuged at 1500×g for 10mins at 4 °C and the supernatant from the first lavage was collected for subsequent cytokine enzyme-linked immunosorbent assay (ELISA) analysis. BAL Cells were counted using a haemocytometer and results expressed as total BAL cell counts (Additional file 2). Cells were adjusted to a concentration of 1 × 10^6^ cells/ml, centrifuged using EZ single cytofunnels (Thermo Scientific, cat# 97200125) onto Superfrost™ Plus slides (Menzeil-Gläser Superfrost Plus, Thermo Scientific, cat# 10149870). Cells were then stained for differential immune cell counts using a Wright-Giemsa stain (Kwik Diff, Thermo Scientific, cat# 9990700). Slides were dried overnight before mounting and counting macrophages, lymphocytes, neutrophils and eosinophils across a minimum of 300 cells per sample. Cells were counted for each animal over three different slides.

### In vitro culture and exposure

Normal human primary bronchial epithelial cells (HPBEC) from 5 different donors without any reported pathology were obtained from Epithelix Sàrl (Switzerland). Cells were cultured at 37 °C in a 5% CO_2_ humidified atmosphere. Cells between passage 2–4 were sub-cultured onto type IV human collagen (Sigma Aldrich, Dorset, UK, Cat# C5533) coated transwell growth supports (Corning, Sigma Aldrich, Dorset, UK, Cat# 3450) to create ALI cultures of epithelial cells. After 4 days of submerged culture in growth media (Epithelix, Cat# EP09AM) to allow cells to reach confluence, apical media was removed from the filter supports. On day 7 after seeding, basolateral media was changed to a differentiation defined media formulation composed of a 50:50 ratio of LHC-9 and DMEM (High Glucose) media, supplemented with bovine serum albumin 1.5 μg/mL, bovine pituitary extract, 180 μg/mL, insulin 0.86 μM, transferrin 0.125 μM, hydrocortisone 1.38 μM, triiodothyronine 0.01 μM, epinephrine 2.7 μM, epidermal growth factor 0.5 ng/mL, retinoic acid 5 × 10^− 8^ M, penicillin G sulphate 100 U/mL, streptomycin sulphate 100 μg/mL, as described [60]. Media change was carried out every 2–3 days and cells were treated between 7 and 12 days post differentiation. For cell treatment, apical deposition of CeO_2_NPs and HDM was carried out in 50 μl of differentiation media.

### Elisa

BAL fluid was analysed for cytokine levels of IL-4 (Cat# DY404), IL-13 (Cat# DY413), CCL11 (Cat# DY420), CCL17 (Cat# DY529), IL-33 (Cat# DY3626), TSLP (Cat# DY555) and CCL20 (DY760) using DuoSet kits from R&D systems (Abingdon, UK) according to manufacturer’s instructions. For mast cell protease 1 (MCPT1) protein detection, lung tissue was homogenised using MagNa lyser beads (Roche, cat# 03358941001) in HBSS lysis buffer containing 2% Triton-× 100 (Sigma Aldrich, cat# 93426-100ML) and protease inhibitor cocktail (Roche, cat# 05892970001). Protein levels of MCPT1 were detected using ELISA as per manufacturer’s instructions (Cat# 88–7503; Invitrogen, VWR Lutterworth, UK). Plasma IgE concentrations were analyzed using a BD OptEIA ELISA kit (cat# 555248; BD Bioscience, Oxford, UK) according to the manufacturer’s instructions. Samples were analyzed in duplicate and diluted as appropriate in sample buffer. Absorbance was assessed at 450 nm with background levels at 570 nm using a plate reader (Bio-Tek Synergy HT). Extrapolation of protein levels was carried out from a standard curve of recombinant proteins.

### ICP-MS measurements

Lung tissue was microwave digested in 20% nitric acid using an UltraWAVE™ microwave digester. Total Ce content was measured using an iCAP Q ICP-MS (Thermo Fisher Scientific, Hemel Hempstead, UK). The ICP-MS was run in KED mode and the isotopes monitored were ^140^Ce and ^63^Cu. Calibration standards (0 – 10 μg/L) were prepared from a Spex CertPrep 50 μg/L stock solution and iridium was used as internal standard. Laser ablation inductively coupled plasma mass spectrometry (LA-ICP-MS) analyses of formalin fixed lung tissue sections was undertaken using a New Wave Research NWR213 laser ablation system (Electro Scientific Industries, Portland, Oregon, USA) linked to the iCAP Q ICP-MS. Laser ablation conditions comprised 5 μm diameter spot size, fluence 10 J/cm^2^, 25μms^− 1^ scan speed and a repetition rate of 20 Hz. Scan log files of ^140^Ce and ^63^Cu track quantities were generated to allow reconstruction of the data into an image using Iolite v3 [61] within Igor Pro 6.36 (Wavemetrics Inc. Oregon, USA).

### RNA extraction and PCR analysis

For in vitro gene expression analysis total RNA was isolated using an RNeasy spin column method (Qiagen, Valencia, CA). For in vivo gene expression, lung tissue (post BAL) was initially homogenised using MagNA lyser bead disruption (Roche Diagnostics, West Sussex, UK) prior to RNeasy spin column based RNA isolation. RNA purity was determined using the nanodrop platform (Thermo Scientific) and cDNA was synthesised using a random hexamer based protocol and reverse transcriptase as per manufacturer’s instructions (Cat# BIO-27036, Bioline Reagents Ltd., UK). SYBR green based real time PCR (40 cycle) was carried out using the QuantStudio™ 6 Flex Real-Time PCR System. Primers were obtained from the published literature or designed using Primer 3 software and provided by Integrated DNA technologies, UK. Primer sequences are listed in the Additional file 2. Cycle thresholds (Ct values) were quantitatively assessed using the delta-delta Ct method and normalized to B-actin control.

### RNA-SEQ and bioinformatic analysis

RNA from lung tissue was analyzed for quality using an Agilent 2100 Bioanalyser and samples with RIN above 8.0 were used for TruSeq™ library preparation (Illumina, San Diego, USA) as previously described [62]. Processing and sequencing was carried out using 90PE sequencing on the Illumina HiSeq™ 2000 platform. Raw sequence data was processed to remove adaptor sequences, contamination and low-quality reads. 20 million clean reads for each sample were brought forward for further processing using CLC Genomics Workbench software (CLCBIO, Aarhus, Denmark). Paired end reads were trimmed to remove remaining adapter and other variable sequences followed by annotation using the GRCm38.p5 mouse reference genome assembly build. For normalization, RPKM (Reads per Kilobase per Million mapped reads) values > 0.5 in at least one treatment condition were selected for differential expression analysis. Further comparative analysis and visualization of differentially regulated transcripts was carried out using Qlucore Omics Explorer software (Qlucore, Lund, Sweden). RNA-SEQ and pathway analysis of differently regulated transcripts was performed using ingenuity pathway analysis (IPA) (Ingenuity Systems, Redwood, CA, USA) and as previously described [62].

### Histochemical and immunohistochemical analysis

After tracheal perfusion fixation of the lung in formalin, the left lung was placed into cassettes (Tissue Tek Processing Embedding cassette, Tissue Tek, cat# 4185TT2), dehydrated and embedded in paraffin wax using the Tissue-Tek system (VIP Sakura). Samples were then fixed into moulds, cut into 5 μm serial sections (Accu-Cut SRM, Sakura), placed onto microscope slides (Menzeil-Gläser Superfrost Plus, Thermo Scientific, Cat# 10149870) before drying overnight at 37 °C. Sections were stained with hematoxylin–eosin (HE) to visualise lung tissue structure and inflammatory infiltrate. Periodic acid–Schiff (PAS) staining was used to determine airway epithelial cell mucin content. Mucin positive cells, identified as pink in colour, were counted in airways of between 100 and 250 μm in diameter. Scoring of airways for mucin positive cells was carried out after counting and expressed as a percentage of total epithelial cells within the airway. A PAS score was then calculated: < 10% = 0 11–20% = 2 21–50% = 3 51–75% = 4 and > 75% = 5. All counts were performed blinded and for 3 airways per individual animal in order to determine a group average. Masson’s trichrome staining of lung sections was used to determine collagen content, reflective of airway fibrosis and remodelling. All histochemical stains were performed using a Sakura Tissue Tek Prisma-E2S (Sakura). Immunohistochemical staining was carried out using the VECTASTAIN Elite ABC Kit (Cat#PK-6100, Vector Laboratories) according to the manufacturer’s instructions using mouse specific antibodies directed against alpha smooth muscle actin (α-sma) (Cat#ab5694, Abcam, Cambridge, UK), proliferating cell nuclear antigen (PCNA) (Cat#13110, Cell Signalling Technology, Leiden, The Netherlands) and chloride channel accessory 1 (CLCA1) (Cat#ab180851, Abcam, Cambridge, UK), at 1:1000, 1:8000 and 1:250 antibody dilutions respectively. Prior to all staining procedures sections were dewaxed and hydrated. This involved first removing the paraffin wax by clearing 3 times with xylene (5 min each), and then rehydrating through an alcohol series from 100% to distilled water (30 s – 1 min in each concentration). Sections were then mounted and a cover slip applied before being imaged using Leica DM 2000 with images captured using a Leica DFC450C camera (Leica Microsystems Ltd).

### Airway mechanics measurements

Mice were anaesthetised by intraperitoneally administration of sodium pentobarbital (90 mg/kg), ensuring the mouse was at surgical levels of anaesthesia throughout the procedure. The mouse was placed under a heat lamp, the trachea exposed and cannulated. Mechanical ventilation was initiated immediately using a computer-controlled piston Flexivent ventilator (Flexivent, Scireq Scientific Respiratory Equipment Inc) system. Prior to airway function measurements, a deep inflation of the lung was performed to recruit closed lung areas and standardize lung volume history. The absence of spontaneous inspiratory efforts was also confirmed using a test pressure volume curve measurement (PVs-V). AHR to inhaled aerosolised methacholine (Acetyl-β-Methylcholine Chloride (cat#A2251-25G, Sigma Aldrich, UK)) (0-100 mg/ml) was then carried out using the forced oscillation technique [63]. All operational scripts for ventilation and force oscillation technique measurements, including data acquisition were handled using Flexiware software (Version 7.6.3).

### Statistical analysis

Statistical significance compared to control values was carried out using one way ANOVA and Fisher’s LSD Test using Graphpad Prism Software. Results are expressed as mean ± standard error of the mean (SEM) unless otherwise stated. Animal measurements were carried out on a minimum of 6 mice per treatment unless otherwise stated.

## Additional files

Additional file 1: **Figure S1.** Total BAL cell counts after repeat CeO2NPs and HDM exposure. Mice (*n* = 5–9 per treatment group) were exposed to CeO2NPs at either low dose (CeLD) (75 μg/kg) or high dose (CeHD) (750 μg/kg) with and without HDM (1.25 mg/kg) according to instillation protocols as described in Fig. 1. Mice were either exposed 9 times over 3 weeks (A) or a single administration for 24 h (B). After treatment, bronchoalveolar cells were analysed for total immune cell content. Results are expressed as mean ± SEM cells counted per ml of BAL fluid. Statistical significance between treatments was carried out by one way ANOVA. Comparisons between particle and HDM treatments alone over control levels are represented as (* *p* < 0.05), while comparisons between particle + HDM combinations and HDM levels are represented as (# *p* < 0.05). **Figure S2.** Effect of CeO2NPs and HDM exposure on primary human airway epithelial cell gene expression. Human primary bronchial epithelial cells (*n* = 5 different donors) in air-liquid interface culture were apically exposed to HDM (1.1 μg/cm2) alone or in combination with CeO2NPs at either 2.2 ng, 67 ng or 1340 ng per cm2, labelled as Ce2.2, Ce67 or Ce1340 respectively. Cells were treated either once for 24 h or 3 x repeat treatments interspersed over 1 week. mRNA was isolated and examined for transcript levels of inflammatory or mucin related gene expression by RT-PCR analysis with results expressed as mean ± SEM fold over control (F.O.C.) levels (A,B). Results were expressed as a heatmap of normalised values with green down and red upregulated expression, where the intensity of colour is proportional to magnitude of change (A). Selected gene expression was also displayed in more detail (B). Statistical significance between treatments was carried out by one way ANOVA. Comparisons between control (CTRL) and HDM treatments are represented as (* *p* < 0.05), while HDM vs HDM + CeO2NP treatments are represented as (# *p* < 0.05). (PPTX 125 kb)

Additional file 2: Primer Sequences. (DOCX 16 kb)

## Abbreviations

α-sma: Alpha smooth muscle actin
AHR: Airway hyperresponsiveness
ALI: Air-liquid interface
BAL: Bronchoalveolar lavage
CeHD: CeO_2_NPs at the higher dose (750 μg/kg)
CeLD: CeO_2_NPs at the lower dose (75 μg/kg)
CeO_2_NP: Cerium dioxide nanoparticle
CeO_2_NPs: Cerium dioxide nanoparticles
CLCA1: Chloride channel accessory 1
Clec10a: C-Type lectin domain containing 10A
CTRL: Control
ELISA: Enzyme-linked immunosorbent assay
HDM: house dust mite
HE: Hematoxylin–eosin
HPBEC: Normal human primary bronchial epithelial cells
Ifit1: Interferon induced protein with tetratricopeptide repeats 1
IL-13: Interleukin-13
IL-25: Interleukin-25
IL-33: Interleukin-33
IL-4: Interleukin-4
IL-5: Interleukin-5
IPA: Ingenuity pathway analysis
Irf7: Interferon Regulatory Factor 7
LA-ICP-MS: Laser ablation inductively coupled plasma mass spectrometry
Ly6i: Lymphocyte antigen 6I
MCPT1: Mast cell protease 1
Muc5AC: Mucin 5AC
PAS: Periodic acid–schiff
PBS: Phosphate buffered saline
Pcna: Proliferating cell nuclear antigen
ROS: Reactive oxygen species
Saa3: Serum Amyloid A3
SD: Standard deviation
SEM: Standard error of the mean
Slc26a4: Solute Carrier Family 26 Member 4
TEM: Transmission electron microscopy
Tslp: Thymic stromal lymphopoietin
