# Supplementary material for: Cerium dioxide nanoparticles exacerbate house dust mite induced type II airway inflammation
Source: Part Fibre Toxicol. 2018 May 23;15:24. doi: 10.1186/s12989-018-0261-5 (PMC5966909; doi:10.1186/s12989-018-0261-5)
Supplement: Supplementary file 1 — Figure S1. Total BAL cell counts after repeat CeO2NPs and HDM exposure. Mice (n = 5–9 per treatment group) were exposed to CeO2NPs at either low dose (CeLD) (75 μg/kg) or high dose (CeHD) (750 μg/kg) with and without HDM (1.25 mg/kg) according to instillation protocols as described in Fig. 1. Mice were either exposed 9 times over 3 weeks (A) or a single administration for 24 h (B). After treatment, bronchoalveolar cells were analysed for total immune cell content. Results are expressed as mean ± SEM cells counted per ml of BAL fluid. Statistical significance between treatments was carried out by one way ANOVA. Comparisons between particle and HDM treatments alone over control levels are represented as (* p < 0.05), while comparisons between particle + HDM combinations and HDM levels are represented as (# p < 0.05). Figure S2. Effect of CeO2NPs and HDM exposure on primary human airway epithelial cell gene expression. Human primary bronchial epithelial cells (n = 5 different donors) in air-liquid interface culture were apically exposed to HDM (1.1 μg/cm2) alone or in combination with CeO2NPs at either 2.2 ng, 67 ng or 1340 ng per cm2, labelled as Ce2.2, Ce67 or Ce1340 respectively. Cells were treated either once for 24 h or 3 x repeat treatments interspersed over 1 week. mRNA was isolated and examined for transcript levels of inflammatory or mucin related gene expression by RT-PCR analysis with results expressed as mean ± SEM fold over control (F.O.C.) levels (A,B). Results were expressed as a heatmap of normalised values with green down and red upregulated expression, where the intensity of colour is proportional to magnitude of change (A). Selected gene expression was also displayed in more detail (B). Statistical significance between treatments was carried out by one way ANOVA. Comparisons between control (CTRL) and HDM treatments are represented as (* p < 0.05), while HDM vs HDM + CeO2NP treatments are represented as (# p < 0.05). (PPTX 125 kb) [file 12989_2018_261_MOESM1_ESM.pptx]

## Slide 1
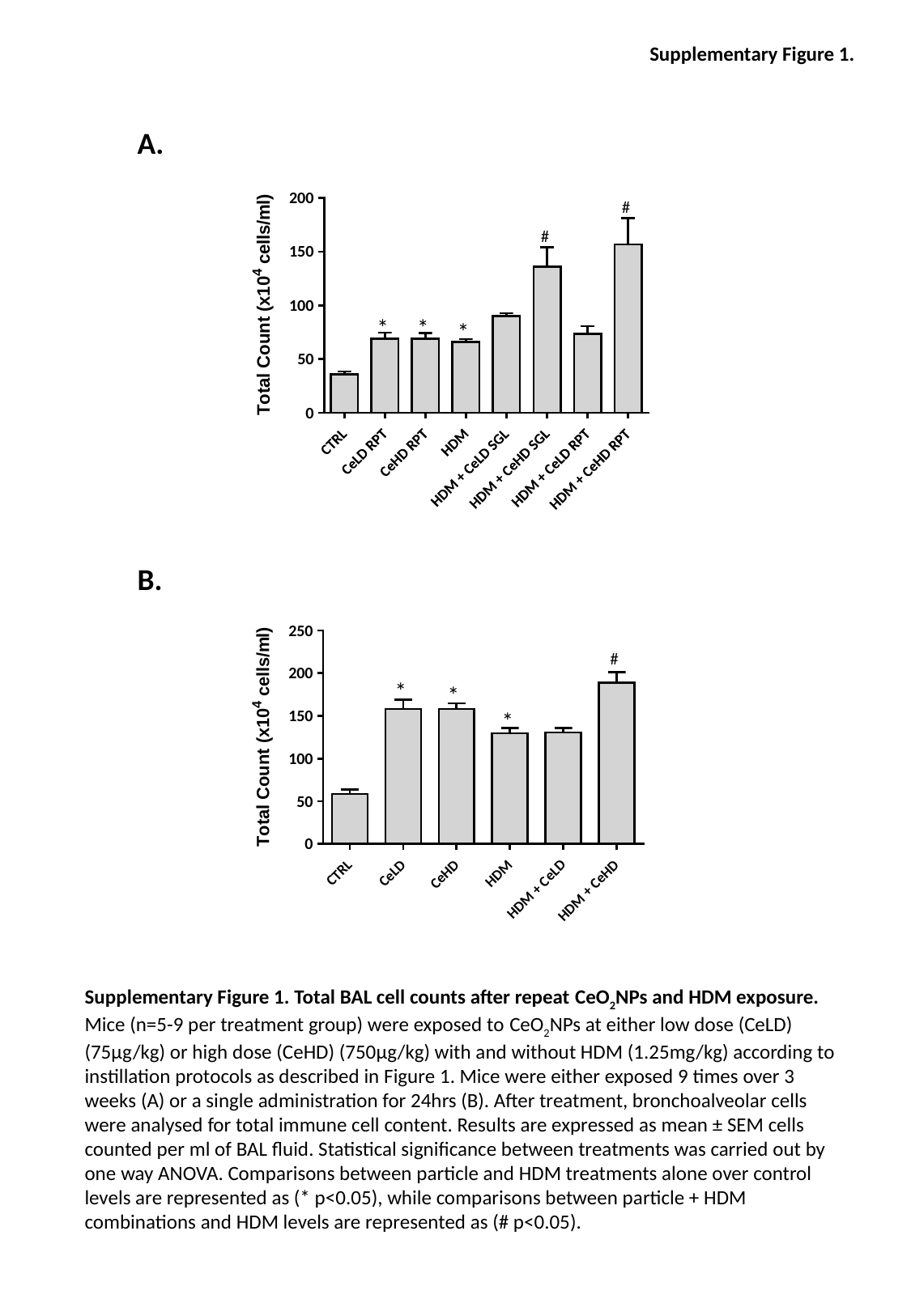

Supplementary Figure 1.
A.
#
#
*
*
*
B.
#
*
*
*
Supplementary Figure 1. Total BAL cell counts after repeat CeO2NPs and HDM exposure.
Mice (n=5-9 per treatment group) were exposed to CeO2NPs at either low dose (CeLD) (75µg/kg) or high dose (CeHD) (750µg/kg) with and without HDM (1.25mg/kg) according to instillation protocols as described in Figure 1. Mice were either exposed 9 times over 3 weeks (A) or a single administration for 24hrs (B). After treatment, bronchoalveolar cells were analysed for total immune cell content. Results are expressed as mean ± SEM cells counted per ml of BAL fluid. Statistical significance between treatments was carried out by one way ANOVA. Comparisons between particle and HDM treatments alone over control levels are represented as (* p<0.05), while comparisons between particle + HDM combinations and HDM levels are represented as (# p<0.05).

## Slide 2
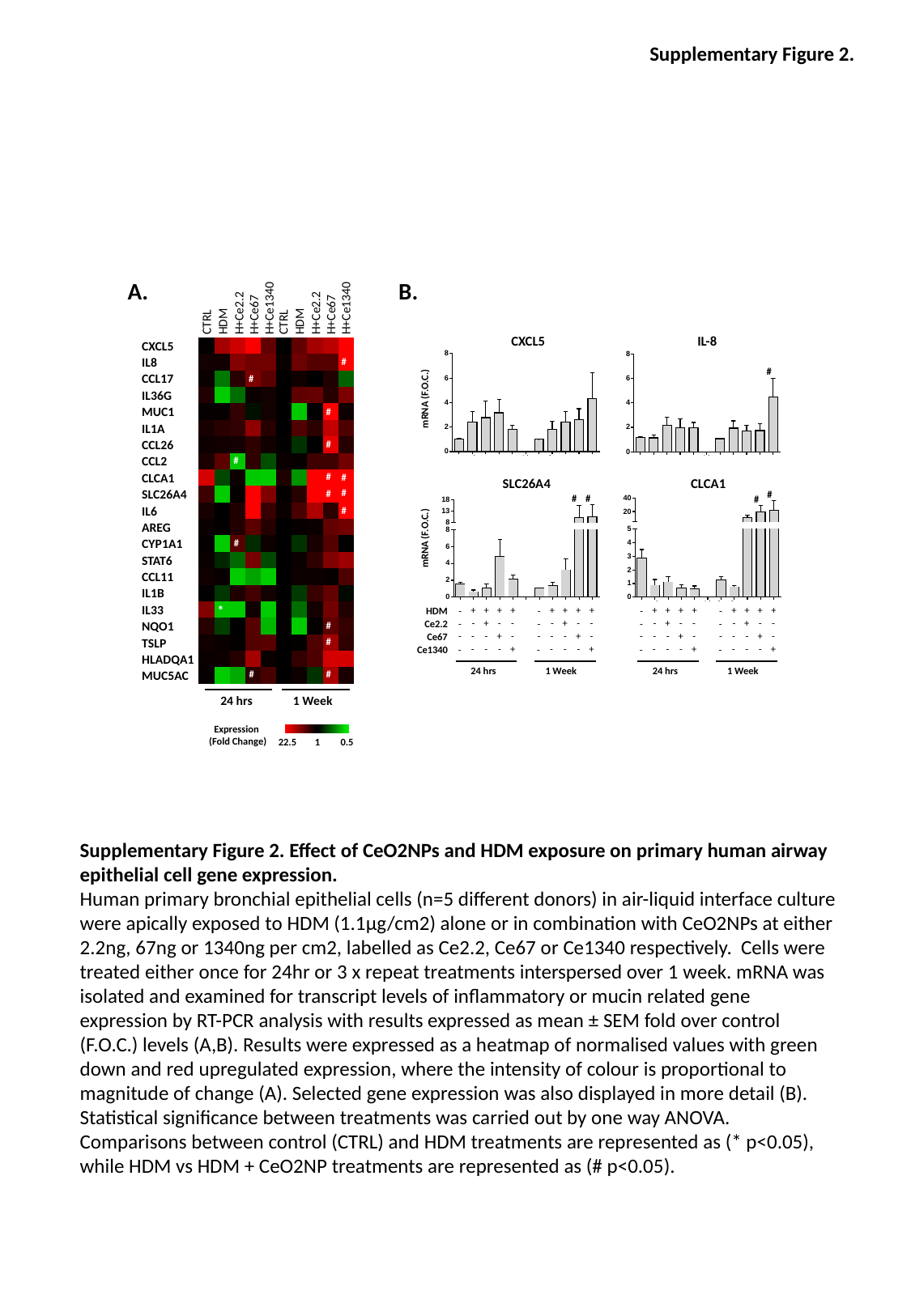

Supplementary Figure 2.
A.
B.
H+Ce2.2
H+Ce67
H+Ce1340
H+Ce2.2
H+Ce67
H+Ce1340
CTRL
HDM
CTRL
HDM
CXCL5
IL-8
| CXCL5 | | | | | | | | | | |
| --- | --- | --- | --- | --- | --- | --- | --- | --- | --- | --- |
| IL8 | | | | | | | | | | |
| CCL17 | | | | | | | | | | |
| IL36G | | | | | | | | | | |
| MUC1 | | | | | | | | | | |
| IL1A | | | | | | | | | | |
| CCL26 | | | | | | | | | | |
| CCL2 | | | | | | | | | | |
| CLCA1 | | | | | | | | | | |
| SLC26A4 | | | | | | | | | | |
| IL6 | | | | | | | | | | |
| AREG | | | | | | | | | | |
| CYP1A1 | | | | | | | | | | |
| STAT6 | | | | | | | | | | |
| CCL11 | | | | | | | | | | |
| IL1B | | | | | | | | | | |
| IL33 | | | | | | | | | | |
| NQO1 | | | | | | | | | | |
| TSLP | | | | | | | | | | |
| HLADQA1 | | | | | | | | | | |
| MUC5AC | | | | | | | | | | |
#
#
#
mRNA (F.O.C.)
#
#
#
#
#
SLC26A4
CLCA1
#
#
#
#
#
#
#
mRNA (F.O.C.)
#
*
+
+
+
+
-
-
+
-
-
-
-
-
+
-
-
-
-
-
+
-
+
+
+
+
-
-
+
-
-
-
-
-
+
-
-
-
-
-
+
-
+
+
+
+
-
-
+
-
-
-
-
-
+
-
-
-
-
-
+
-
+
+
+
+
-
-
+
-
-
-
-
-
+
-
-
-
-
-
+
-
HDM
Ce2.2
#
Ce67
#
Ce1340
24 hrs
1 Week
24 hrs
1 Week
#
#
24 hrs
1 Week
Expression
(Fold Change)
22.5
1
0.5
Supplementary Figure 2. Effect of CeO2NPs and HDM exposure on primary human airway epithelial cell gene expression.
Human primary bronchial epithelial cells (n=5 different donors) in air-liquid interface culture were apically exposed to HDM (1.1µg/cm2) alone or in combination with CeO2NPs at either 2.2ng, 67ng or 1340ng per cm2, labelled as Ce2.2, Ce67 or Ce1340 respectively. Cells were treated either once for 24hr or 3 x repeat treatments interspersed over 1 week. mRNA was isolated and examined for transcript levels of inflammatory or mucin related gene expression by RT-PCR analysis with results expressed as mean ± SEM fold over control (F.O.C.) levels (A,B). Results were expressed as a heatmap of normalised values with green down and red upregulated expression, where the intensity of colour is proportional to magnitude of change (A). Selected gene expression was also displayed in more detail (B). Statistical significance between treatments was carried out by one way ANOVA. Comparisons between control (CTRL) and HDM treatments are represented as (* p<0.05), while HDM vs HDM + CeO2NP treatments are represented as (# p<0.05).
