# Supplementary material for: Cerium dioxide nanoparticles exacerbate house dust mite induced type II airway inflammation
Source: Part Fibre Toxicol. 2018 May 23;15:24. doi: 10.1186/s12989-018-0261-5 (PMC5966909; doi:10.1186/s12989-018-0261-5)
Supplement: Supplementary file 2 — Primer Sequences. (DOCX 16 kb) [file 12989_2018_261_MOESM2_ESM.docx]

**Additional file 2**

**Primer Sequences**

*Mouse*

| **Gene Name (Forward / Reverse)** | **Sequence (5' - 3')** |
| --- | --- |
| SLC26A4-Ms-F | GGAGCAGTGTGGGTTCTTTG |
| SLC26A4-Ms-R | GGAAGCAAGTCTACGCATGG |
| MMP12-Ms-F | TTCCGTCGTGTCACCAAAAC |
| MMP12-Ms-R | TGTGTGACCTGTACACCTCC |
| CCL17-Ms-F | AGGTCACTTCAGATGCTGCT |
| CCL17-Ms-R | GAAACACGATGGCATCCCTG |
| CD177-Ms-F | TCTCACGAAAGGCTGCACTA |
| CD177-Ms-R | TCAGAAAAGCAGAGAGGGCA |
| B-actin-Ms-F | CCTCTATGCCAACACAGTGC |
| B-actin-Ms-R | CCTGCTTGCTGATCCACATC |
| CCL11-Ms-F | GTCACTTCCTTCACCTCCCA |
| CCL11-Ms-R | TTCTTGGGGTCAGCACAGAT |
| CLCA1-Ms-F | TCCGAAGAACAGAGCCATGT |
| CLCA1-Ms-R | GACAGGGTGGAAAGAGGTCA |
| CXCL5-Ms-F | CTCCTCCAGCATATCCCGAG |
| CXCL5-Ms-R | TAAGCAAACACAACGCAGCT |
| IL13-Ms-F | TTGAAAGGCTGGGTATCGGT |
| IL13-Ms-R | AGCCAGCTCCTCATGAATGT |
| IL4-Ms-F | AACGAGGTCACAGGAGAAGG |
| IL4-Ms-R | TCTGCAGCTCCATGAGAACA |
| IL5-Ms-F | GATCCTCCTGCCTCCTCTTC |
| IL5-Ms-R | AGACCCTGATGCAACGAAGA |
| MUC5AC-Ms-F | CTTTCCCAACACACCACTGG |
| MUC5AC-Ms-R | GTGAGGTGGGATGTGTGGTA |
| MCPT1-Ms-F | CCTAGAAGCTCACCAAGGCT |
| MCPT1-Ms-R | AAACCCACCACATCTGTCCT |

*Human*

| **Gene Name (Forward / Reverse)** | **Sequence (5' - 3')** |
| --- | --- |
| AREG-F | GTGGTGCTGTCGCTCTTGATA |
| AREG-R | ACTCACAGGGGAAATCTCACT |
| B-actin-F | TCATGAAGTGTGACGTGGACATC |
| B-actin-R | CAGGAGGAGCAATGATCTTGATCT |
| IL1A-F | GGCGTTTGAGTCAGCAAAGAAG |
| IL1A-R | CATGGAGTGGGCCATAGCTT |
| IL1B-F | CCGACCACCACTACAGCAAGG |
| IL1B-R | GGGCAGGGAACCAGCATCTTC |
| NQO1-F | AGGACCCTTCCGGAGTAAGA |
| NQO1-R | TCAGTTGGGATGGACTTGCC |
| HLA-DQA1-F | CCGCTGCTACCAATGAGGTT |
| HLA-DQA1-R | TGTTGACCACAGGAGGAAAGATG |
| CYP1A1-F | CACCATCCCCCACAGCAC |
| CYP1A1-R | ACAAAGACACAACGCCCCTT |
| MUC5AC-F | TGTGGCGGGAAAGACAGC |
| MUC5AC-R | CCTTCCTATGGCTTAGCTTCAGC |
| TSLP-F | CCAGGCTATTCGGAAACTCA |
| TSLP-R | CGCCACAATCCTTGTAATTG |
| IL6-F | AAGCCAGAGCTGTGCAGATGAGTA |
| IL6-R | TGTCCTGCAGCCACTGGTTC |
| IL33 -F | CAAAGAAGTTTGCCCCATGT |
| IL33-R | AAGGCAAAGCACTCCACAGT |
| IL8-F | TTGGCAGCCTTCCTGATTTC |
| IL8-R | AACTTCTCCACAACCCTCTG |
| CCL17-F | AGACATCTGAGGACTGCTCCAG |
| CCL17-R | ATCTCCCTCACTGTGGCTCTTC |
| CCL2-F | GCTCAGCCAGATGCAAT |
| CCL2-R | GCTTGTCCAGGTGGTCCATG |
| CCL26-F | AACTCCGAAACAATTGTGACTCAGCTG |
| CCL26-R | GTAACTCTGGGAGGAAACACCCTCTCC |
| CXCL5-F | TGTTTACAGACCACGCAAGG |
| CXCL5-R | TTGTTTCCACCGTCCAAAAT |
| IL36G-F | GAGCTGGGTGGTATAAGGCT |
| IL36G-R | GTGCTAGTCATGCCATCGTG |
| STAT6-1F | GCGGCTCTATGTCGACTTTC |
| STAT6-1R | GGTGCTGGACAGTGTCTGAA |
| CCL11-F | AGGAGAATCACCAGTGGCAAA |
| CCL11-R | GGAATCCTGCACCCACTTCTT |
